# The CCAAT/enhancer binding protein (C/EBP) δ is differently regulated by fibrillar and oligomeric forms of the Alzheimer amyloid-β peptide

**DOI:** 10.1186/1742-2094-8-34

**Published:** 2011-04-14

**Authors:** Veronica Ramberg, Linda M Tracy, Malin Samuelsson, Lars NG Nilsson, Kerstin Iverfeldt

**Affiliations:** 1 Department of Neurochemistry, Stockholm University, SE-10691 Stockholm; Sweden; 2 Department of Public Health and Caring Sciences, Uppsala University, SE-75185 Uppsala, Sweden

## Abstract

**Background:**

The transcription factors CCAAT/enhancer binding proteins (C/EBP) α, β and δ have been shown to be expressed in brain and to be involved in regulation of inflammatory genes in concert with nuclear factor κB (NF-κB). In general, C/EBPα is down-regulated, whereas both C/EBPβ and δ are up-regulated in response to inflammatory stimuli. In Alzheimer's disease (AD) one of the hallmarks is chronic neuroinflammation mediated by astrocytes and microglial cells, most likely induced by the formation of amyloid-β (Aβ) deposits. The inflammatory response in AD has been ascribed both beneficial and detrimental roles. It is therefore important to delineate the inflammatory mediators and signaling pathways affected by Aβ deposits with the aim of defining new therapeutic targets.

**Methods:**

Here we have investigated the effects of Aβ on expression of C/EBP family members with a focus on C/EBPδ in rat primary astro-microglial cultures and in a transgenic mouse model with high levels of fibrillar Aβ deposits (tg-ArcSwe) by western blot analysis. Effects on DNA binding activity were analyzed by electrophoretic mobility shift assay. Cross-talk between C/EBPδ and NF-κB was investigated by analyzing binding to a κB site using a biotin streptavidin-agarose pull-down assay.

**Results:**

We show that exposure to fibril-enriched, but not oligomer-enriched, preparations of Aβ inhibit up-regulation of C/EBPδ expression in interleukin-1β-activated glial cultures. Furthermore, we observed that, in aged transgenic mice, C/EBPα was significantly down-regulated and C/EBPβ was significantly up-regulated. C/EBPδ, on the other hand, was selectively down-regulated in the forebrain, a part of the brain showing high levels of fibrillar Aβ deposits. In contrast, no difference in expression levels of C/EBPδ between wild type and transgenic mice was detected in the relatively spared hindbrain. Finally, we show that interleukin-1β-induced C/EBPδ DNA binding activity to both C/EBP and κB sites is abolished after exposure to Aβ.

**Conclusions:**

These data suggest that both expression and function of C/EBPδ are dysregulated in Alzheimer's disease. C/EBPδ seems to be differently regulated in response to different conformations of Aβ. We propose that Aβ induces an imbalance between NF-κB and C/EBP transcription factors that may result in abnormal responses to inflammatory stimuli.

## Background

Alzheimer's disease (AD) is a neurodegenerative disorder and is the most common cause of dementia among the elderly. Accumulation of amyloid-β (Aβ) peptides in the brain is considered to be a key step in the pathogenesis of the disease and leads to formation of amyloid plaques in brain parenchyma. The Aβ peptides can be truncated at both the C- and N-terminal ends, and also undergo posttranslational modifications. Although Aβ1-40 (40 amino acids long) is the most abundant form, the major focus is on Aβ1-42 which is more prone to aggregate and considered to be the most neurotoxic form. Aβ is found in different aggregation states in the brain ranging from monomers and non-fibrillar aggregates, termed oligomers, to a highly fibrillar form found in the deposits. Recent evidence suggests that diffusible Aβ oligomers have the most toxic properties [1,2]. However, it should also be noted that Aβ fibril-containing senile plaques precede the development of dystrophic neurites [3] and of spinodendritic calcium decompartmentalization that presumably leads to cognitive dysfunction [4]. In addition to massive neurodegeneration, chronic neuroinflammation is a pathological hallmark of AD, manifested by activated microglia and reactive astrocytes. Accumulation and deposition of Aβ can trigger activation of glial cells, which will set off an inflammatory response that, over time, becomes chronic causing a persistent deleterious condition [5].

The role of neuroinflammation in the development and progression of AD is, however, not clear. Neuroinflammation is often referred to as a "double-edged sword". On the one hand microglia and astrocytes secrete inflammatory cytokines, chemokines and neurotoxins upon activation, and can thereby promote neuronal degeneration. On the other hand, activated microglia surrounding Aβ plaques may have beneficial effects by phagocytosis of, and thus elimination of, Aβ [6]. Astrocytes have also been reported to be able to migrate towards Aβ plaques and, upon contact, to degrade Aβ [7,8]. This somewhat confusing picture calls for delineation of signaling pathways that may be involved in the beneficial effects of neuroinflammation or that may promote neurodegeneration.

The inflammatory response is, to a large degree, orchestrated by the transcription factor nuclear factor κB (NF-κB). However, NF-κB works in concert with other transcription factors. Of particular interest are members of the CCAAT/enhancer binding protein (C/EBP) family that can amplify the effects of NF-κB and may also form heteromeric complexes with NF-κB [9-11]. C/EBP is a protein family consisting of six members, C/EBPα-ζ (reviewed in [12]). In order to be active, C/EBPs will form homo- or heterodimers with each other or with other transcription factors. Until recently, C/EBP studies have mainly focused on the liver, where these proteins regulate expression of a variety of genes including acute phase proteins [13]. However, C/EBPα, β and δ are also all expressed in brain [14,15]. Among their target genes are many pro-inflammatory cytokines including interleukin- (IL-) 6 (an early inflammatory marker in AD brain), inducible nitric oxide synthase, complement factors, and cyclooxygenase-2 (COX-2) [16]. In both brain and liver, inflammatory stimuli have been shown to, in general, down-regulate expression of C/EBPα and up-regulate expression of C/EBPβ and δ [15]. Several previous studies have suggested that members of the C/EBP transcription factor family are dysregulated in AD and in response to Aβ. mRNA levels of C/EBPα, β and δ have all been shown to be up-regulated in hippocampus of AD patients [17-19] and protein levels of C/EBPδ have also been reported to be up-regulated in AD brain [20]. However, in a recent study we observed that C/EBPδ DNA binding activity to a C/EBP motif is, instead, completely blocked in IL-1β-stimulated primary astro-microglial cultures from rats after exposure to oligomeric forms of Aβ peptides [21]. It can be speculated that Aβ peptides cause an imbalance between NF-κB and C/EBP transcription factors that could result in abnormal responses to inflammatory stimuli. To further investigate how the inflammatory response in the AD brain may be affected we have analyzed the effects of different aggregation states of Aβ on NF-κB-C/EBP cross-talk, and also C/EBPα and δ expression levels in activated primary glial cultures. In addition, we have analyzed the expression levels of C/EBPα, β and δ and in mice with high levels of fibrillar Aβ deposits.

## Methods

### Primary astro-microglial cultures and treatments

Primary mixed glial cultures consisting of 5-10% microglia and 90-95% astrocytes (hereafter referred to as astro-microglial cultures) were obtained from male Sprague Dawley pups less than 24 h of age as previously described [22]. The experimental procedures were approved by Stockholm North Local Committee on Ethics of Animal Experiments and performed in accordance with international standards on animal welfare. Cells were seeded in 60 mm culture dishes and maintained in DMEM glutamax containing 10% fetal bovine serum (FBS) and 0.1% penicillin-streptomycin (PEST) for 20-22 days (all from GIBCO). Medium was exchanged every 3-4 day. 18 h prior to experiment, medium was exchanged to DMEM glutamax containing 0.2% FBS and 0.1% PEST. Confluent cells were exposed to 10 μM Aβ(1-42), 10 μM Aβ(42-1) (American peptide), 10 ng/ml rat recombinant IL-1β (Biosource) and/or 1 μg/ml lipopolysaccharide (LPS, Sigma) for 3 h before analysis. Aβ preparations enriched in oligomeric or fibrillar forms were prepared as previously described [23,24]. Briefly, Aβ was dissolved in hexafluoroisopropanol (HFIP) to a final concentration of 1 mM. HFIP was then removed by centrifugation under vacuum, and Aβ was then resuspended in dimethyl sulfoxide (DMSO, Sigma) to a final concentration of 5 mM. To obtain oligomers, culture medium was added to the peptide to a final concentration of 100 μM followed by an incubation at 4°C for 24 h. For a fibril-enriched preparation, 10 mM HCl was added to the peptide to a final concentration of 100 μM, followed by incubation for 24 h at 37°C. The different Aβ preparations were analyzed using western blot (see western blot) and Thioflavin T assay (T3516, Sigma). In the Thioflavin T assay, Aβ samples were analyzed in phenol red-free medium and according to the manufacturer's instructions.

### Nuclear extracts

Nuclear extracts were prepared as follows: cells were washed in ice-cold PBS, scraped and subsequently centrifuged for 2 min. Thereafter the pellet was washed again in PBS, centrifuged for 25 s and resuspended in HB buffer (10 mM Tris HCl pH 7.4, 10 mM KCl, 1.5 mM MgCl_2_, 0.5 mM PMSF, 0.5 mM β-mercaptoethanol), centrifuged for 25 s and resuspended in lysis buffer (HB buffer, 0.4% Nonidet-40). Samples were then lysed on ice for 10 min. Nuclear fractions were pelleted by centrifugation for 5 min, and then resuspended in buffer C (10 mM HEPES pH 7.9, 0.4 M NaCl, 1 mM EDTA, 1 mM DTT, 1 mM PMSF) and thereafter placed on a shaking platform for 30 min and centrifuged for 5 min. The supernatant containing nuclear extract was collected and stored at -80°C for approximately 24 h before use. All steps were performed at 4°C and all centrifugations were performed at 16,000 g.

### Transgenic mice

Transgenic mice, tg-ArcSwe, carrying both the Artic (E693G) and the Swedish (KM670/671NL) APP double mutations were originally of C57Bl/6-CBA-F1 background [25], but then bred on a C57Bl/6 background for more than six generations (incipient congenics). The experiments were approved by an ethical committee (Uppsala djurförsöksetiska nämnd) and performed in compliance with national and local animal care and use guidelines (protocol #C223/8). Animals were 15.5-17 month of age when sacrificed. At this age tg-ArcSwe mice have previously been shown to possess a plaque burden of approximately 4%, as measured by Aβ immunostaining of tissue sections followed by quantitative image analysis [26,27]. Mice were anesthetized with 0.3 ml Avertin (25 mg/ml) and intracardially perfused with 0.9% saline solution. The brains were removed and dissected into amyloid-containing forebrain (including cortex and hippocampus) and amyloid-sparse hindbrain (cerebellum and brainstem) (cf. [25]). After dissection the samples were snap-frozen and stored in liquid nitrogen until use. As a control for changes related to age and Aβ deposition in the animals, 4 month-old tg-ArcSwe and non-transgenic animals were also investigated. Protein lysates were prepared as follows: forebrain and hindbrain were homogenized in ice-cold RIPA buffer (50 mM Tris HCl pH 8, 150 mM NaCl, 1% Nonidet-40, 0.5% sodium deoxycholate, 1% SDS, 1 mM PMSF) to a concentration of 25 mg/ml and placed on a shaking platform for 5 h at 4°C. Thereafter the samples were centrifuged for 20 min at 9,500 g and the protein containing supernatant was collected and stored at -80°C until used.

### Aβ ELISA

For measurement of total Aβ levels, ninety-six-well plates were coated with 50 ng/well of the N-terminal Aβ-specific antibody 82E1 (IBL-Hamburg, Hamburg, Germany) in PBS and blocked with 1% BSA in PBS with 0.15% Kathon (Rohm & Haas, Philadelphia, PA). Brain tissues homogenized in RIPA-buffer were supplemented with formic acid (to a final concentration of 70%), neutralized with 1 M Tris (pH 10) and diluted in ELISA incubation buffer (PBS with 0.1% BSA and 0.05% Tween-20). Samples or standard (recombinant Arctic Aβ1-40), were treated similarly, and added to the plates in duplicates and incubated for 2 h at room temperature with shaking. Biotinylated mAb27 (1 μg/ml), with a conformation-dependent epitope in the mid-domain of Arctic Aβ [28], was used to measure total Aβ. The biotinylated detection antibody as well as streptavidin-horseradish peroxidase (SA-HRP; diluted 1:2000; Mabtech, Nacka, Sweden) was allowed to incubate for 1 h in successive steps. K-blue aqueous (ANL-Produkter, Älvsjö, Sweden) was used as HRP-substrate, the reaction was stopped with 1 M H_2_SO_4 _and the optical density was measured at 450 nm with a SpectraMax 190 (Molecular Devices, Sunnyvale, CA). Wells were washed three times between each step in ELISA washing buffer (PBS with 0.1% Tween-20 and 0.15% Kathon).

### Western blot

Protein concentrations were estimated using Bicinchoninic acid assay (BCA, Pierce). Protein lysates (20 μg/lane from primary astro-microglial cultures and 40 μg/lane from transgenic mice tissues) were mixed with Laemmli sample buffer and boiled for 5 min. Proteins were separated on 12% SDS-polyacrylamide gels and thereafter blotted onto polyvinylidene diflouride (PVDF) membranes (Amersham Pharmacia). For analysis of oligomer- and fibril-enriched Aβ preparations, samples were not preheated and they were run on a 12% SDS-polyacrylamide gel without stacking gel. The membranes were incubated in PBS containing 5% milk, 0.1% Tween-20 (Bio-Rad) and 0.1% BSA (Sigma) for 2 h at room temperature to block unspecific binding, and thereafter incubated in the above-mentioned PBS solution with primary antibody overnight at 4°C. Antibody concentrations were as follows: C/EBPα ((14AA)X) and C/EBPδ ((C-22)X, Santa Cruz Biotechnologies) 1:4000 for primary astro-microglial cultures and 1:1000 for transgenic samples, β-actin (A-2066, Sigma) 1:5000 and 6E10 (ab49682, Abcam) raised against Aβ(1-17) 1:2000. Next, membranes were washed in PBS containing 2.5% milk and 0.1% Tween-20 for 4 × 15 min and thereafter incubated with secondary antibody HRP coupled anti-rabbit IgG or anti-mouse IgG (GE Healthcare) 1:5000, diluted in the same solution, for 1 h at room temperature. Finally, membranes were washed 4 × 15 min in PBS containing 0.1% Tween-20. Blots were incubated in ECL Plus reagents (GE Healthcare) for 5 min and exposed to Hyper film (GE Healthcare). Membranes exposed to anti-C/EBPβ ((C19)X, Santa Cruz Biotechnologies) were first washed 5 min in TBS and rinsed 10 s in methanol [29]. Thereafter the membranes were allowed to dry before overnight incubation at 4°C with primary antibody, 1:500, diluted in TBS containing 5% milk and 0.05% Tween-20. Membranes were then washed 2 × 15 s in TBS containing 0.05% Tween-20 and incubated for 1 h at RT with secondary antibody (HRP coupled anti-rabbit IgG) 1:5000, diluted in TBS containing 5% milk and 0.05% Tween-20. Finally, membranes were washed 2 × 30 min in TBS containing 0.05% Tween-20, incubated in ECL Plus reagents for 5 min and exposed to Hyper film.

### Electrophoretic mobility shift assay (EMSA)

Protein concentrations were established using BCA assay. EMSA was performed as previously described [21,30] with minor modifications. Briefly, C/EBP sense oligonucleotide 5'-TGCAGATTGCGCAATCTGCA-3 (MWG) was labeled with γ-^32^P dATP (Perkin Elmer) using T4 polynucleotide kinase (Fermentas). Nuclear extracts (5 μg) from primary astro-microglial cultures were incubated with anti-C/EBPδ ((C-22) X, Santa Cruz Biotechnologies) for 30 min at room temperature prior to binding reaction with ^32^P-labeled probe. Samples were separated on a 5% polyacrylamide gel at 150 V for 1 h. Gels were exposed to a phosphorimager screen overnight.

### Streptavidin-agarose pull-down assay

C/EBPδ binding to the κB DNA element was analyzed by streptavidin-agarose pull-down [31,32]. Single stranded biotinylated κB sense oligonucleotide 5'-AGTTGAGGGGACTTTCCCAGGC-3' and antisense oligonucleotide 5'-GCCTGGGAAAGTCCCCTCAACT-3' (MWG), were hybridized by incubation at 100°C for 1 h and then allowed to cool down slowly (for approx. 30 min) to room temperature. Nuclear extracts (150 μg) from primary astro-microglial cultures were mixed with streptavidin-agarose bead suspension (according to the manufacturer's recommendation; S1638, Sigma) and 4 μg of double stranded biotinylated oligonucleotides in 500 μl of buffer B2 (PBS, 1 mM EDTA, 1 mM DTT, protease inhibitor cocktail Complete (Roche Diagnostics)). The mixture was then placed on a shaking platform for 2 h at room temperature. Thereafter the samples were centrifuged at 550 g for 1 min and the pellet washed 3 times in buffer B2. Finally the pellet was dissolved in 35 μl 2x Laemmli sample buffer, incubated at 100°C for 5 min and centrifuged at 7000 g for 30 s and the supernatant was collected. Samples were loaded on a 12% SDS-polyacrylamide gel and analyzed by western blot. As a control for unspecific binding to the agarose beads, identical κB oligonucleotides lacking biotin were used.

### Statistical analysis

Data was analyzed using student's t-test or one-way ANOVA followed by Tukey's post hoc test.

## Results

### Fibril-enriched Aβ preparations down-regulate levels of C/EBPδ in activated primary astro-microglial cells

**Figure 1:**
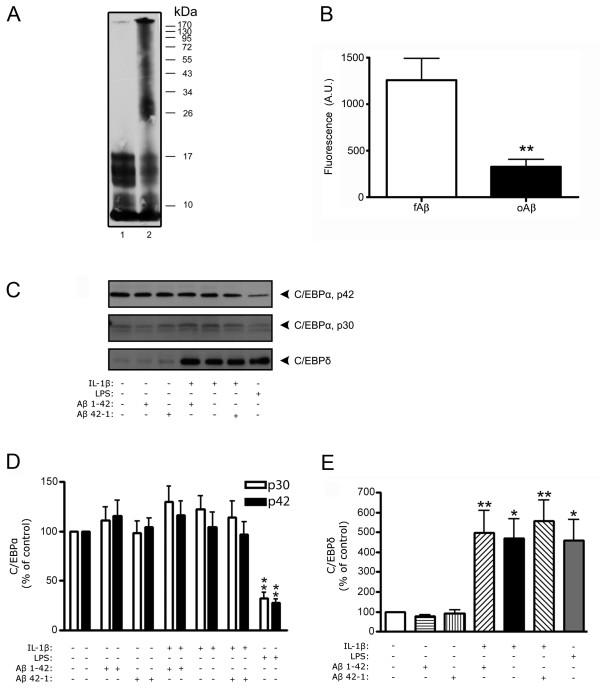
**Oligomer-enriched Aβ has no effect on IL-1β-induced C/EBPδ expression levels in rat primary astro-microglial cultures**. A) Representative western blot of Aβ preparations used for cell treatments showing the presence of high levels of fibrillar Aβ only in the fibril-enriched preparation (lane 2) and not in the oligomeric-enriched preparation (lane 1). B) Statistical analysis of Thioflavin T fluorescence in the presence of fibril-enriched Aβ preparation (fAβ; open bar) and oligomer-enriched Aβ preparation (oAβ; filled bar). The data represent mean ± SEM for four independent experiments. A.U., arbitrary units. C) Representative western blot of C/EBPα (p42 and p30) and C/EBPδ in primary astro-microglial cultures from rat. Cells were exposed to an Aβ preparation mainly consisting of oligomers for 3 h. 20 μg of protein lysate was loaded in each lane. D) Relative abundance of C/EBPα showing decreased protein levels in response to LPS (1 μg/ml), but no effect in response to oligomer-enriched Aβ (10 μM) and/or IL-1β (10 ng/ml). E) Relative abundance of C/EBPδ showing increased protein levels in response to IL-1β or LPS, and no change on response to IL-1β when oligomer-enriched Aβ is present. The data represent mean ± SEM for two cultures derived from three independent experiments. *p < 0.05, **p < 0.01, significantly different as indicated.

**Figure 2:**
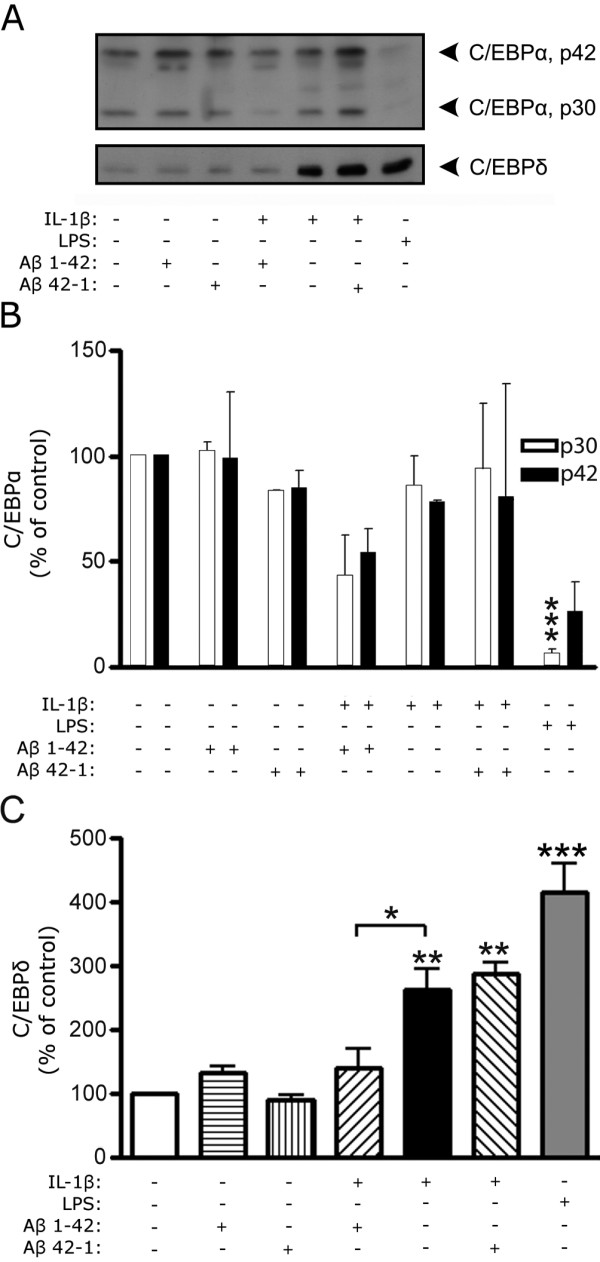
**Fibril-enriched Aβ inhibits IL-1β-induced C/EBPδ expression in rat primary astro-microglial cultures**. A) Representative western blot of C/EBPα (p42 and p30) and C/EBPδ in primary astro-microglial cultures from newborn rat. Cells were exposed to an Aβ preparation highly enriched in fibrils for 3 h. 20 μg of protein lysate was loaded in each lane. B) Relative abundance of C/EBPα showing significantly decreased protein levels in response to LPS (1 μg/ml), but no significant effect in response to fibril-enriched Aβ (10 μM) or IL-1β (10 ng/ml). C) Relative abundance of C/EBPδ showing increased levels in response to IL-1β or LPS, and decreased C/EBPδ levels after exposure to IL-1β together with Aβ compared to treatment with IL-1β alone. The data represent mean ± SEM for two cultures derived from three independent experiments. *p < 0.05, **p < 0.01, ***p < 0.001, significantly different from control or otherwise indicated.

The effect of Aβ on expression levels of C/EBPα and δ in primary astro-microglial cells was analyzed by western blot. Two different preparations of Aβ1-42 were used: one containing high levels of oligomers and one containing high levels of fibrils. The different Aβ preparations were analyzed using western blot and Thioflavin T assay (Figure 1A,B). Cells were treated with Aβ enriched in oligomers or fibrils in the presence or absence of IL-1β (10 ng/ml) for 3 h prior to harvesting and preparing nuclear extracts. IL-1β, which is known to be induced in AD brains, was used to activate the cells. For comparison, cells were also treated with LPS (1 μg/ml). Two different isoforms of C/EBPα; p30 and p42, which were both significantly down-regulated by LPS treatment, were detected (Figure 1C,D and 2A,B). This is consistent with earlier findings in primary glial cultures showing that C/EBPα is mainly regulated through toll-like receptors [33]. Neither Aβ nor IL-1β alone had any effect on the levels of the two isoforms of C/EBPα. However, it should be noted that the levels of p30 and p42 appeared to be reduced when cell cultures were stimulated with fibril-enriched Aβ preparations together with IL-1β although this effect was not statistically significant.

Both IL-1β and LPS induced up-regulation of C/EBPδ (Figure 1C,E and 2A,C). Aβ enriched in oligomers (Figure 1C,E) or fibrils (Figure 2A,C) did not have any significant effect on basal C/EBPδ levels. However, treatment with Aβ containing high levels of fibrils did significantly decrease IL-1β-induced C/EBPδ levels by ~75% (Figure 2A,C). Neither Aβ preparations devoid of fibrils nor the reversed peptide Aβ42-1 (prepared as the Aβ1-42), used as a control, had any effect on IL-1β-stimulated up-regulation of C/EBPδ (Figure 1C,E and 2A,C).

### C/EBPδ levels are decreased in brain areas of tg-ArcSwe mice rich in amyloid plaques

**Figure 3:**
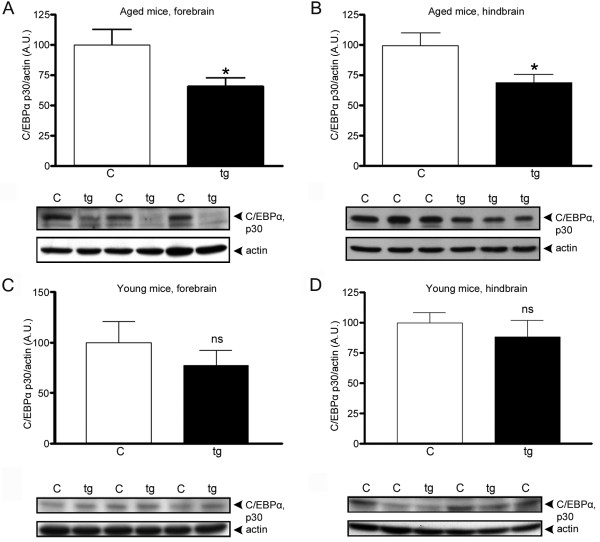
**Decreased levels of C/EBPα also in brain areas with low Aβ plaque burden from aged tg-ArcSwe mice**. Relative abundance and representative western blot of C/EBPα (p30) in forebrain (A) and pathology-spared hindbrain (B) from 15.5-17 month-old tg-ArcSwe mice (tg) and non-transgenic mice (c). The results show a decrease in protein levels in both brain areas indicating that the observed down-regulation does not directly relate to Aβ plaque burden. The data represent mean ± SEM for six transgenic and six control mice. Young transgenic mice did not show significantly altered expression in forebrain (C) or hindbrain (D). The data represent mean ± SEM for three transgenic and five control mice. 40 μg of protein lysate was loaded in each lane and β-actin was used as a loading control. *p < 0.05, significantly different from control. A.U., arbitrary units.

**Figure 4:**
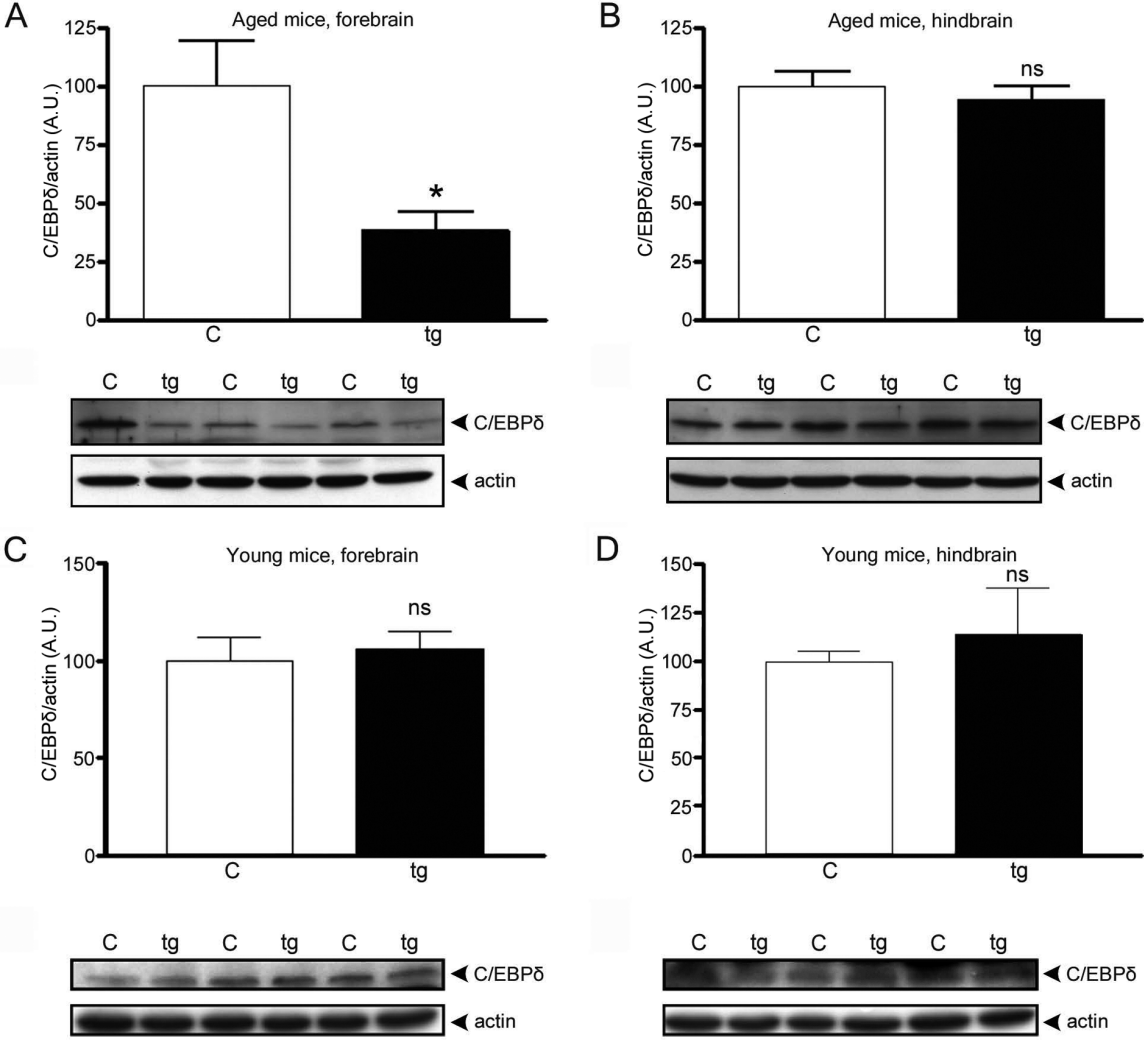
**Decreased levels of C/EBPδ in brain areas with high Aβ plaque burden from aged tg-ArcSwe mice**. Relative abundance and representative western blot of C/EBPδ from forebrain (A) of aged tg-ArcSwe mice (tg) and non-transgenic mice (c) showing a decrease in protein levels in amyloid-rich areas whereas the relatively Aβ plaque-free hindbrain (B) does not show altered expression. The data represent mean ± SEM for six transgenic and six control mice. Young transgenic mice did not show alterations in C/EBPδ expression compared to non-transgenic littermates in forebrain (C) or hindbrain (D). The data represent mean ± SEM for three transgenic mice and five control mice. 40 μg of protein lysate was loaded in each lane and β-actin was used as a loading control. *p < 0.05, significantly different from control. A.U., arbitrary units.

**Figure 5:**
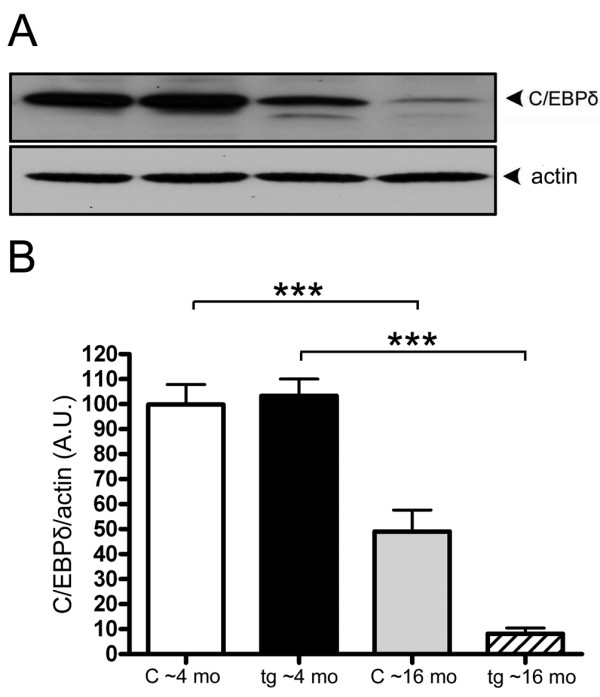
**C/EBPδ levels are decreased with ageing and Aβ plaque formation**. Relative abundance (A) and representative western blot (B) of C/EBPδ from forebrain of young and aged tg-ArcSwe mice (tg) and non-transgenic littermates (c). When comparing C/EBPδ levels between young and aged mice, the results indicate an overall C/EBPδ decrease not only in response to Aβ but also to age. The data represent mean ± SEM for five transgenic mice and six control mice. 40 μg of protein lysate was loaded in each lane and β-actin was used as a loading control. ***p < 0.001, significantly different as indicated. A.U., arbitrary units.

**Figure 6:**
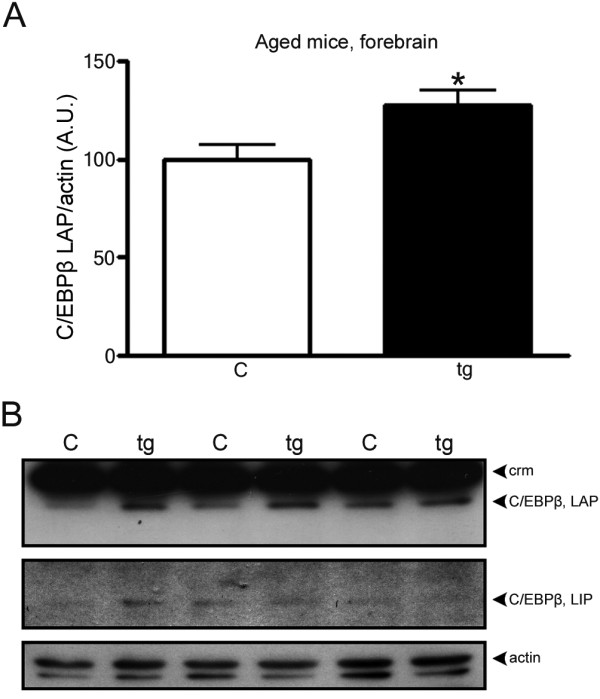
**Increased levels of C/EBPβ in brain areas with high Aβ plaque burdens from aged tg-ArcSwe mice**. A) Relative abundance of C/EBPβ showing increased protein levels in forebrain of aged tg-ArcSwe mice (tg) compared to non-transgenic mice (c). B) Representative western blot showing two isoforms of C/EBPβ (LAP and LIP). Only the C/EBPβ LAP isoform was quantified. The data represent mean ± SEM for six transgenic and six control mice. 40 μg of protein lysate was loaded in each lane and β-actin was used as a loading control. *p < 0.05, significantly different from control; crm, cross-reactive material (cf., [53,54]). C/EBPβ protein levels in hindbrain from aged mice and from both forebrain and hindbrain from young mice were below or close to detection limit. A.U., arbitrary units.

Based on these results, the effects of Aβ fibrils *in vivo *were investigated. Tg-ArcSwe mice (15.5-17-months-old), with both the Artic (E693G) and Swedish (KM670/671NL) APP double mutations, were chosen since aged animals exhibit an abundance of Aβ fibrils [26]. A significant down-regulation of C/EBPα was found in both forebrain and hindbrain of aged tg-ArcSwe mice. C/EBPα p30 levels were ~31-33% lower compared with age-matched non-transgenic littermates (Figure 3A,B). Unfortunately the effect on p42 could not be determined due to co-migration of strong nonspecific bands on the western blots. In contrast to a previous report of findings in human AD patient tissue [20], the levels of C/EBPδ were decreased ~62% in forebrain of aged tg-ArcSwe mice compared with age-matched non-transgenic mice (Figure 4A). Total Aβ levels in forebrain from these aged tg-ArcSwe mice were 227 ± 48 ng Aβ/mg tissue (mean ± SEM, N = 6) and were below the detection limit (i.e., <20 pg Aβ/mg tissue) in non-transgenic mice. Our findings are consistent with the results from IL-1β-activated astro-microglial cultures treated with fibrilar Aβ (Figure 2A,C). Analysis of the relatively Aβ plaque-free hindbrain showed a down-regulation of C/EBPα p30 levels similar to that found in forebrain (Figure 3B) indicating that this is not a local Aβ plaque-dependent phenomenon. In contrast, no difference in C/EBPδ levels was observed in hindbrain when transgenic mice and non-transgenic age-matched littermates were compared (Figure 4B). To ensure that the changes in levels of C/EBPs were due to age-dependent accumulation of Aβ and formation of Aβ fibrils, and not some unrelated effect of the transgene, brain tissue from young tg-ArcSwe mice with low Aβ load were analyzed and compared with age-matched non-transgenic mice. These experiments showed that there are no significant differences between tg-ArcSwe mice and non-transgenic mice with respect to C/EBPα p30 (Figure 3C,D) and C/EBPδ levels (Figure 4C,D). However, direct comparison of non-transgenic and tg-ArcSwe mice of different ages indicated that C/EBPδ levels are down-regulated both in response to age and Aβ plaque burden (Figure 5A,B). The C/EBPδ levels in forebrain were significantly decreased in aged non-transgenic mice by ~51% and further decreased in aged tg-ArcSwe mice by ~92% compared to young animals. In contrast, the LAP isoform (~35 kDa) of C/EBPβ was significantly up-regulated (~27% higher) in the forebrain of aged tg-ArcSwe mice as compared to age-matched non-transgenic control (Figure 6A,B). C/EBPβ LAP levels were below or close to detection limit in the hindbrain of aged mice and in both forebrain and hindbrain of young animals and could therefore not be quantified.

### IL-1β-increased binding activity of C/EBPδ is blocked by fibril-enriched Aβ preparations

**Figure 7:**
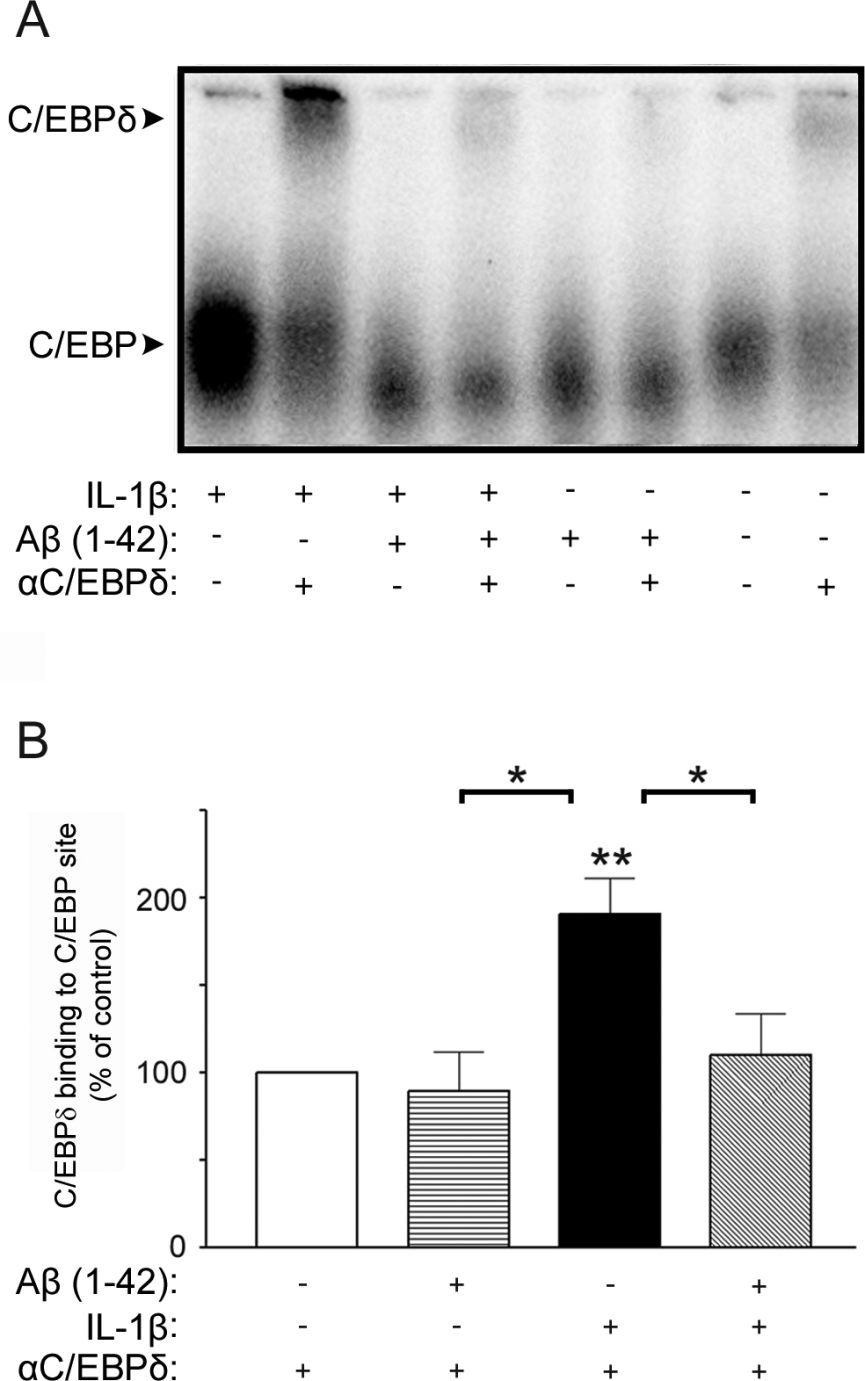
**C/EBPδ binding activity induced by IL-1β is blocked by Aβ fibrils**. A) Representative EMSA showing inhibition of IL-1β- (10 ng/ml) induced C/EBPδ binding activity in primary astro-microglial cultures exposed to fibril-enriched Aβ (10 μM) for 3 h. B) Relative abundance of C/EBPδ binding activity showing that fibril-enriched Aβ inhibits IL-1β-induced C/EBPδ binding, but has no effect on its own. The data represent mean ± SEM for two cultures derived from three to six independent experiments. *p < 0.05, **p < 0.01, significantly different from control or otherwise indicated.

To further investigate the effects of fibrillar forms of Aβ, EMSA was performed to analyze C/EBPδ binding activity. Primary astro-microglial cells were treated for 3 h with Aβ fibrils in the presence or absence of IL-1β prior to harvesting. The results show that C/EBPδ binding to double-stranded oligonucleotides containing a C/EBP binding site is down-regulated by ~89% in cultures concomitantly treated with IL-1β and fibril-enriched Aβ preparations compared to cells treated with IL-1β alone (Figure 7A,B). The down-regulation in DNA binding activity reflects the down-regulation in protein level.

### C/EBPδ binding to a κB site induced by IL-1β is inhibited by Aβ

**Figure 8:**
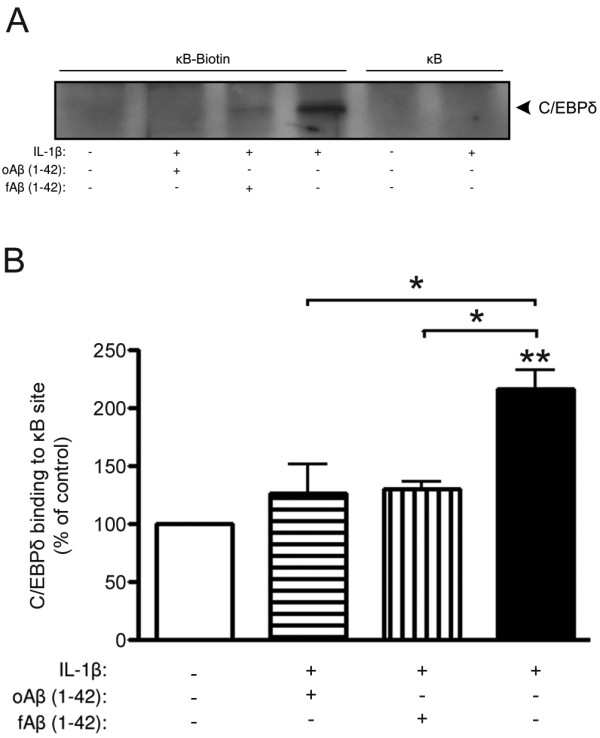
**IL-1β-induced C/EBPδ binding to a κB site is inhibited by Aβ**. A) Representative western blot of C/EBPδ after streptavidin-agarose pull-down using biotinylated κB oligonucleotides. Primary astro-microglial cells were exposed to oligomer- or fibril-enriched preparations of Aβ (10 μM, oAβ or fAβ) in combination with IL-1β (10 ng/ml) for 3 h. κB oligonucleotides without biotin were used as a control for unspecific binding. B) Relative levels of C/EBPδ binding to the κB site showing that IL-1β induces a significant increase that is inhibited after Aβ exposure. The data represent mean ± SEM for three independent experiments. *p < 0.05, **p < 0.01, significantly different from control or otherwise indicated.

In order to test the hypothesis that C/EBPδ can shift its preferable DNA binding site and binding partners, and to analyze the effects of Aβ, C/EBPδ binding to a κB response element was investigated. Nuclear extracts from primary astro-microglial cultures treated for 3 h with IL-1β together with Aβ were analyzed in streptavidin-agarose pull-down experiments. Cells treated with IL-1β showed increased binding to the κB element compared to untreated control cells. In the presence of fibril-enriched Aβ, IL-1β-induced binding of C/EBPδ to the κB site was completely blocked (Figure 8A,B). We also analyzed the effect of oligomer-enriched preparations of Aβ. Surprisingly, the oligomeric conformation of Aβ decreased C/EBPδ binding to the κB site to the same extent as the fibrillar form in IL-1β activated cells (Figure 8A,B).

## Discussion

In this study we observed that IL-1β-induced expression of C/EBPδ was inhibited in rat primary astro-microglial cultures after exposure to Aβ. However, this effect is dependent on the presence of fibrillar forms of Aβ and no effect on protein levels was observed after exposure to an oligomer-enriched Aβ preparation. In general, C/EBPδ is up-regulated in response to inflammatory stimuli. Our results instead indicate an Aβ-dependent decrease in C/EBPδ levels. This decrease of C/EBPδ was further confirmed using an AD transgenic mouse model, tg-ArcSwe mice, characterized by high levels of fibrillar Aβ deposits [25]. When analyzing brain areas of aged mice carrying a high Aβ load, C/EBPδ was significantly down-regulated compared to the same brain areas from aged non-transgenic littermates. This effect on C/EBPδ levels is not the expected result since up-regulation of inflammatory markers has been detected in other AD transgenic mice models (cf., [34]). An Aβ-induced inflammatory response in tg-ArcSwe mice is, however, supported by down-regulated C/EBPα levels and up-regulated C/EBPβ levels in forebrain from aged tg-ArcSwe mice.

Differential effects of oligomeric and fibrillar forms of Aβ on glial cells have been reported earlier: Rat astro-microglial cultures show significantly higher levels of inflammatory markers, such as tumor necrosis factor (TNF)-α, when exposed to oligomeric compared to fibrillar Aβ [35]. In microglial cells TNF-α induction is typical for a classic cytotoxic phenotype, whereas an alternative activation state is not correlated with expression of inflammatory cytokines and is instead characterized by increased Aβ phagocytic capabilities. A recent study in a *PS1xAPP *transgenic AD model showed that hippocampal microglial cells switch from the alternative activation state to the classical cytotoxic phenotype during aging [36]. Interestingly, only microglia not located in the vicinity of Aβ plaques express this classical cytotoxic phenotype in 18-month-old transgenic mice. Microglial cells surrounding plaques are instead TNF-α negative. The same authors also showed that both oligomeric Aβ and soluble extract from 18-month-old transgenic mouse hippocampus produces potent TNF-α induction in astro-microglial cultures from non-transgenic mice [36]. One possibility is that increased expression of C/EBPδ is a marker for classical cytotoxic glial cells and that the fibrillar form of Aβ actually blocks induction of this phenotype. This could also explain the decreased levels of C/EBPδ that we observed in brain areas with high Aβ load in aged tg-ArcSwe mice, since the Arctic mutation not only increases the rate of Aβ protofibril formation but also fibril formation and senile plaque deposition [26,37].

As indicated above, increased levels of C/EBPβ and δ are expected when an inflammatory response is induced. It is believed that during inflammation the rapidly and transiently activated NF-κB pathway is crucial for a primary wave of gene induction and that a second and more long-term wave of gene transcription is mediated by other transcription factors including the C/EBP family (cf., [12,38,39]). Members of the C/EBP family can form heterodimers with NF-κB subunits [9,11]. Previous studies have indicated a reciprocal cross-coupling between NF-κB and C/EBPs: NF-κB and C/EBPs seem to synergistically activate promoters with C/EBP sites, and inhibit promoters with κB motifs [10,38,40]. In this study we show that, in astro-microglial cells, IL-1β induces binding of C/EBPδ-containing complexes to a κB site. It could be speculated that this is a part of a negative feed-back mechanism regulating the effect of NF-κB activation. If this is true, the inhibition of C/EBPδ binding to the κB motif that we observe after exposure to Aβ could result in prolonged effects of NF-κB acting at this site.

When the astro-microglial cells were exposed to fibril-enriched Aβ preparations the IL-1β-induced binding of C/EBPδ to both the C/EBP and the κB motifs were more or less abolished. This could be expected since the IL-1β-induced increase of C/EBPδ protein levels was strongly reduced by Aβ fibrils. However, the effect of oligomer-enriched Aβ preparations on C/EBPδ binding activity was not expected. Although no effect could be observed on protein levels, IL-1β-induced binding of C/EBPδ to the κB site was decreased to the same degree as after treatment with Aβ fibrils. In addition, we showed in a previous study that oligomer-enriched Aβ preparations also decrease the binding of C/EBPδ to the C/EBP site [21]. Taken together these results indicate that oligomer-enriched Aβ affects the functional properties of C/EBPδ rather than expression levels.

The activity of C/EBPs has been shown to be dependent upon phosphorylation. Phosphorylation can occur in a negative regulatory domain of C/EBPs and is believed to relieve the inhibitory effect on their DNA binding and transactivation domains [41,42]. C/EBPβ has a conserved MAPK consensus phosphorylation motif [43]. Phosphorylation induced by the Ras pathway upon inflammatory stimuli will lead to a switch from a repressed to an active conformation [43-45]. In murine embryonic fibroblasts, LPS-induced expression of both C/EBPβ and δ is dependent on NF-κBp65 and IκB kinase (IKK) 2 [46]. In cells lacking the IKK-related kinase IKKi, LPS will still activate NF-κB and induce C/EBPβ and δ expression, but C/EBPδ-specific DNA binding is absent [46].

IL-1β, as well as other inflammatory stimuli, has been shown to activate the Ras pathway [47]. The Ras pathway in turn can result in both extracellular signal-regulated kinase (ERK) 1/2 and IKKi activation [48]. A recent study showed that LPS-induced upregulation of C/EBPδ in a microglial cell line could be prevented by an ERK inhibitor [31]. Both LPS-induced C/EBPδ protein expression and DNA binding to a C/EBP motif have been reported to be blocked by a combination of MAP kinase inhibitors (ERK/JNK/p38 inhibitors) [49]. Interestingly, an age-dependent and significant reduction of the active form of ERK1 (phosphoERK1; pERK1) has been observed in cerebral cortex from the AD-transgenic mouse model tg2576/PS1^P264L ^[50]. In addition, in the tg2576 mice model, which shows a slower build-up of amyloid burden, a slight, but significant, increase of pERK2 is observed in CA1 of hippocampus at 13 months of age. This is followed by a significant reduction at 20 months of age [51]. Thus, it is possible that our results showing decreased levels of C/EBPδ both in astro-microglial cultures exposed to Aβ fibrils and in aged tg-ArcSwe mice could be explained by reduced MAP kinase activity.

Our results showing decreased levels of C/EBPδ both in astro-microglial cultures exposed to Aβ fibrils and in aged tg-ArcSwe mice seems contradictory to a previous study from Rogers' laboratory showing up-regulated C/EBPδ in AD brains [20]. However, as discussed above, data from transgenic mouse models indicate that the effect on C/EBPδ may correlate with a later phase when build-up of fibrillar Aβ deposits reaches a certain level that will lead to reduced expression of C/EBPδ. It may be assumed that the ratio Aβ fibrils:oligomers may be lower in brain tissue from patients with the sporadic form of AD as compared to aged tg-ArcSwe transgenic mice. However, it cannot be ruled out that DNA binding and transcriptional activity is reduced also in AD brain since we propose that oligomer-enriched Aβ disturbs the functional properties of C/EBPδ rather than expression levels.

The C/EBP family members exert pleiotropic effects in tissues in which they are expressed and are also expressed in a number of different cell types. C/EBPα, β, and δ are all expressed in primary astro-microglial cultures. At least in human AD brains, C/EBPδ immunoreactivity seems to localize primarily to astrocytes [20]. However, in brain tissue from mice neuronal localization of C/EBPs has also been reported [20,52] and this may also explain the high levels of C/EBPδ in young mice. At this point we have not identified the cell types which are responsible for the effects on tissue levels of C/EBPα, β, and δ in tg-ArcSwe mouse brain. It is also possible that the differential effects on the different isoforms (i.e., decreased levels of C/EBPα in both affected and non-affected brain areas, increased levels of C/EBPβ in affected areas and decreased levels of C/EBPδ only in affected areas) mirror the effects of Aβ deposits on different cell types.

## Conclusions

Our results indicate that different aggregation states of Aβ affect the transcription factor C/EBPδ differently during inflammatory conditions. Aβ oligomers, on the one hand, seem to have an effect on C/EBPδ function, as shown by reduced DNA binding activity induced by IL-1β, rather than affecting C/EBPδ protein levels. Aβ fibrils, on the other hand, reduce both protein expression levels and binding activity to C/EBP and κB sites. *In vivo *studies using aged plaque-depositing tg-ArcSwe mice further confirm down-regulation of C/EBPδ caused by the presence of Aβ fibrils. We propose that AD pathology causes an imbalance between NF-κB and C/EBP transcription factors that may result in abnormal responses to inflammatory stimuli. However, the functional consequences of a disturbed C/EBPδ signaling in AD remain to be determined.

## Competing interests

The authors declare that they have no competing interests.

## Authors' contributions

The original design of this study was made by VR, MS, KI. VR coordinated the experiments. VR and LT performed the western blot analyses. EMSA was performed by LT. Streptavidine-agarose pull-down assay and Thioflavin T assays were performed by VR. LNGN maintained the colony of transgenic mice, prepared and dissected brain tissues and performed Aβ ELISA. LNGN and LT participated in the design of the study. VR, LT and KI wrote the first draft of the manuscript. All authors discussed the results and commented on the manuscript. All of the authors have read and approved the final version of the manuscript.
